# Challenges in diagnosing paediatric malaria in Dar es Salaam, Tanzania

**DOI:** 10.1186/1475-2875-12-228

**Published:** 2013-07-03

**Authors:** Gro EA Strøm, Christel G Haanshuus, Maulidi Fataki, Nina Langeland, Bjørn Blomberg

**Affiliations:** 1 Department of Clinical Science, University of Bergen, Bergen, Norway; 2 National Centre for Tropical Infectious Diseases, Department of Medicine, Haukeland University Hospital, Bergen, Norway; 3 Muhimbili University of Health and Allied Sciences, Dar es Salaam, Tanzania; 4 Muhimbili National Hospital, Dar es Salaam, Tanzania

**Keywords:** Malaria, Diagnostics, Polymerase chain reaction, Blood microscopy, Rapid diagnostic test, Tanzania, Paediatrics, Fever

## Abstract

**Background:**

Malaria is a major cause of paediatric morbidity and mortality. As no clinical features clearly differentiate malaria from other febrile illnesses, and malaria diagnosis is challenged by often lacking laboratory equipment and expertise, overdiagnosis and overtreatment is common.

**Methods:**

Children admitted with fever at the general paediatric wards at Muhimbili National Hospital (MNH), Dar es Salaam, Tanzania from January to June 2009 were recruited consecutively and prospectively. Demographic and clinical features were registered. Routine thick blood smear microscopy at MNH was compared to results of subsequent thin blood smear microscopy, and rapid diagnostics tests (RDTs). Genus-specific PCR of *Plasmodium* mitochondrial DNA was performed on DNA extracted from whole blood and species-specific PCR was done on positive samples.

**Results:**

Among 304 included children, 62.6% had received anti-malarials during the last four weeks prior to admission and 65.1% during the hospital stay. Routine thick blood smears, research blood smears, PCR and RDT detected malaria in 13.2%, 6.6%, 25.0% and 13.5%, respectively. Positive routine microscopy was confirmed in only 43% (17/40), 45% (18/40) and 53% (21/40), by research microscopy, RDTs and PCR, respectively. Eighteen percent (56/304) had positive PCR but negative research microscopy. Reported low parasitaemia on routine microscopy was associated with negative research blood slide and PCR. RDT-positive cases were associated with signs of severe malaria. Palmar pallor, low haemoglobin and low platelet count were significantly associated with positive PCR, research microscopy and RDT.

**Conclusions:**

The true morbidity attributable to malaria in the study population remains uncertain due to the discrepancies in results among the diagnostic methods. The current routine microscopy appears to result in overdiagnosis of malaria and, consequently, overuse of anti-malarials. Conversely, children with a false positive malaria diagnosis may die because they do not receive treatment for the true cause of their illness. RDTs appear to have the potential to improve routine diagnostics, but the clinical implication of the many RDT-negative, PCR-positive samples needs to be elucidated.

## Background

Malaria is a major cause of paediatric morbidity and mortality in many developing countries, particularly in sub-Saharan Africa [1,2].

In many areas where malaria is endemic it is highly overdiagnosed [3]. Febrile illness can have many causes, especially in children, who are more susceptible to infections and who present with more diffuse symptoms than adults [4]. Differentiation of fever caused by malaria, septicaemia and other diseases is challenging due to similar clinical presentation and poor diagnostic facilities. Furthermore, there is increasing evidence that malaria predisposes for invasive bacterial disease, making this differentiation even more complex [5].

Overworked health-workers in understaffed institutions may contribute to the overdiagnosis and overtreatment of malaria [6,7]. Furthermore, malaria remains a major diagnosis in febrile patients in many endemic areas, despite evidence from research studies of decreasing numbers of verifiable malaria cases [8]. Recent estimates suggest that malaria mortality in children under five years of age in Tanzania has dropped from 60,880 in 2000 to 26,606 in 2010 [9].

Blood smear microscopy, the long-time gold standard for malaria diagnostics, is increasingly supplemented or replaced by rapid diagnostic tests (RDTs) [10]. Lack of trained and experienced personnel, poor microscopes and lack of staining reagents and other equipment can lead to unreliable microscopy results [6]. Studies done on the implementation of RDTs have shown promising results [11] and may contribute to reduce overtreatment of malaria and to halt the development of anti-malarial resistance [12].

Polymerase chain reaction (PCR) is a reliable technique for identifying malaria cases [13]. In some developed countries it has been tested as a routine diagnostic method [14], but it is generally only used in research and to confirm other laboratory findings.

With a diminishing number of cases and deaths attributable to malaria, the World Health Organization (WHO) recommends anti-malarial treatment to be based on laboratory results, when available, also in children [15]. The current study was performed to assess the morbidity and mortality attributable to malaria among febrile children admitted to Muhimbili National Hospital (MNH), and to compare malaria diagnostics by microscopy, RDT and PCR.

## Methods

### Study site and study population

Dar es Salaam is a major city located in a malaria-endemic area in coastal Tanzania, but may have lower rates of malaria transmission in its urban core [16]. MNH is a tertiary hospital receiving referrals from all over Tanzania as well as a large number of local patients. The predominant malaria species transmitted in the area is *Plasmodium falciparum*[17]. In mainland Tanzania there were 5,48 million presumed and confirmed cases of malaria in 2011 [2]. The prevalence of malaria among children six months to five years of age in 2011–2012, irrespective of symptoms, was 3,6% by RDT and 0,3% by thick blood smear microscopy in the Dar es Salaam region [18].

Informed, written consent was obtained from the child’s parent or guardian by signature or thumbprint. Children between one month and seven years of age, for whom consent was obtained, were recruited consecutively from January to June 2009 at the paediatric wards A and B at MNH. Inclusion criteria were admission due to chief complaint of fever or measured fever (temperature ≥ 37.5°C, axilla) on admission [19]. A primary investigator recruited patients and collected samples and clinical data assisted by ward staff.

A research permit was obtained from the Tanzanian Commission for Science and Technology (COSTECH), and ethical clearance was received from the appropriate bodies at MUHAS and MNH and from the Regional Ethical Committee in Norway. The study was done in collaboration between MUHAS/MNH and the University of Bergen/Haukeland University Hospital, Norway.

### Data collection

Upon admission, a thick blood smear was obtained from each child for routine malaria investigation at MNH. An additional thin blood smear was prepared, and stored for subsequent staining and assessment. A vial of venous blood with EDTA was collected from each child. As a part of hospital routine, a full blood cell count was performed on the EDTA-blood. The remaining content was stored at −20°C for ensuing DNA extraction. Blood slides and EDTA blood vials were transferred to the University of Bergen using the Material Transfer Agreement of Muhimbili University of Health and Allied Sciences (MUHAS) and the EDTA blood samples were kept frozen using dry ice during transport.

A case report form was completed for each child by questioning the parent/guardian and consulting the child′s medical file. Date of birth, gender, mother′s level of education, and whether the child was referred from another hospital, slept under a mosquito net at home, had permanent residence in Dar es Salaam, had known sickle cell disease, had convulsions, and how long the child had been ill, were recorded. Whether treatment with anti-malarials and antibiotics had been given and whether the child had travelled outside Dar es Salaam during the previous four weeks before admission were also documented. Upon admission, weight, axillar temperature, respiratory rate (tachypnea defined as ≥ 60/min if age < 2 months, ≥ 50/min if age two to eleven (<12) months, ≥ 40/min if age one to seven years [20]), pulse/heart rate (tachycardia defined as >160 beats/min if age <12 months, >120/min if age one to seven years [20]), spleno- and hepatomegaly, abdominal distention, jaundice, palmar pallor, and level of consciousness, were recorded for each child. The results of haematological parameters (haemoglobin (Hb), platelet count, erythrocyte count, leucocyte total and differential count), blood slide for malaria, and blood culture with species identification and antibacterial drug susceptibility testing were recorded, if done. Any treatment with antibiotics and anti-malarials during stay at the hospital, and length of hospital stay were recorded along with the outcome, dichotomized as dead or alive. Re-admissions during the study period were followed to observe final patient outcome.

### Malaria detection using blood slides

Thick blood smears taken in routine malaria diagnostics at MNH were stained, with Fields stains A and B, and examined according to hospital routine. Parasitaemia of positive slides were quantified as number of parasites seen per 200 white blood cells. Parasitaemia measured as parasites per μl was calculated using the leucocyte count of each case.

Thin blood smears (research slides) were stained with Giemsa 5% for 20 min after 30-sec fixation with 100% methanol. One hundred high-power microscopic fields were examined before a slide was declared negative. Parasitaemia of positive slides was reported in percentage of infected red blood cells. To quantify parasites per μl, 1% parasitaemia was assumed to be 50,000 parasites per μl according to Hänscheid *et al.*[21]. All slides were examined once and a second experienced microscopist reviewed all slides with uncertain results. Both microscopists were blinded to PCR, RDT and routine thick smear microscopy results.

### Malaria detection using PCR

DNA was extracted from 200 μl whole blood (venous blood collected in a vial with EDTA anti-coagulant) using QIAamp DNA Blood Mini Kit (Qiagen, Hilden, Germany) as described by the manufacturer, and eluted in a final volume of 100 μl.

A genus-specific PCR targeting *Plasmodium* mitochondrial genome, as described by Haanshuus *et al.*[22], but with a primer concentration of 1 μM, was performed on DNA extracted from whole blood. Amplification was done using GeneAmp PCR System 9700 (Applied Biosystems, Carlsbad, CA, USA).

All samples positive by genus-specific PCR were analysed using a *P. falciparum, Plasmodium vivax, Plasmodium ovale* and *Plasmodium malariae* species-specific PCR protocol applying primers targeting 18S as previously published by Padley *et al.*[23]. A modified version of the protocol using four separate reaction mixtures was used, as described by Haanshuus *et al.*[22].

Analysis was done by electrophoresis using 2% SeaKem™ agarose gel (Lonza, Rockland, ME, USA) with 1X GelRed™ (Biotium, Hayward, CA, USA).

DNA sequencing in one direction, as described by Haanshuus *et al.*[22], was done on PCR products that were positive by genus-specific PCR but negative by species-specific PCR.

### Malaria detection using RDT

Retrospectively, samples of remaining EDTA-blood stored at −20°C (from 258 of included patients) were tested with the RDT First Response Malaria Ag pLDH/HRP2 Combo card test following the manufacturer’s instructions. The test detects histidine-rich protein 2 (HRP2) for *P. falciparum* and lactate dehydrogenase (pLDH) for all species (PAN; *P. falciparum, P. vivax, P. ovale* and *P. malariae*) on two separate bands. This RDT has among the best panel detection scores for combo tests on the WHO Rapid Diagnostic Test Performance Round 4 [24].

### Statistical methods

Statistical analysis was done using IBM SPSS Statistics version 19 (SPSS Inc, IBM Company). Differences were reported as statistically significant if p-values were < 0.05. Categorical variables were assessed with the Chi-squared test or Fisher′s exact test for analyses with few observations. Odds ratio was calculated for each variable.

Multivariate analysis was performed to evaluate potential confounding among the clinical variables analysed as risk factors associated with a diagnosis of malaria by the various test methods. It was done by automated and manual backwards stepwise logistic regression on all variables obtaining P < 0.2 in the univariate analysis. The multivariate analysis for predictors of positive RDT excluded the following factors with extensive numbers of missing values: very low weight for age, referral from other hospital, no antibiotics within the previous four weeks, tachycardia for age, and no tachypnea for age.

## Results

### Characteristics of participants

Among a total of 1,443 children admitted at the general paediatric wards during the study period, 469 fit the inclusion criteria and consented to participate. Among study subjects for whom consent was obtained, routine thick blood slide, research thin blood smear and EDTA-blood for PCR were obtained from 304 children, and these are the subjects included in the further analyses. Two-thirds of participants were referrals from other hospitals.

The mean age of the study participants was 22.1 months and 175 (57.6%) were males. Mean axillar temperature upon admission was 38.4°C. During hospital stay, 22.7% died. There was no significant association between fatal outcome and positive PCR (21.7% case fatality rate), research microscopy (4.3%) or RDT (10.3%) as shown in Additional file 1. There was no significant difference in case fatality rates between PCR-positive cases that received anti-malarials and those that did not .

Within the four weeks prior to admission, 74.5% had received antibiotics and 62.6% had received anti-malarials, 83.8% of these had been treated with recommended anti-malarials (artemisinin combination therapy (ACT), or quinine). Children under 12 months of age were more likely to have received pre-treatment with anti-malarials than older children (p = 0.038). No significant association was found between pre-treatment with anti-malarials and PCR and microscopy results. During hospital admission, 65.1% received anti-malarial treatment and 96.1% received antibiotics.

### Laboratory and clinical results

While 40/304 (13.2%) of the routine thick blood smears examined at MNH were positive, only 20/304 (6.6%) of the thin research blood smears were positive. Genus-specific PCR was positive for 76/304 (25.0%) patients. All PCR-positive samples were identified as *P. falciparum* by species-specific PCR (55) or DNA sequencing (21) and none as *P. vivax, P. malariae* or *P. ovale.*

Positive routine microscopy was confirmed in only 43% (17/40), 45% (18/40) and 53% (21/40), by research microscopy, RDTs and PCR, respectively. All 20 positive research thin blood smears were PCR-positive (Table 1).

**Table 1:** Concordance of PCR with routine and research microscopy and RDT results

		**Routine microscopy**		**Research microscopy**		**RDT**	
		**Pos (N = 40)**	**Neg (N = 264)**	**Pos (N = 20)**	**Neg (N = 284)**	**Pos (N = 36)**	**Neg (N = 222)**
**PCR**	**pos (%)**	21 (52.5%)	55 (20.8%)	20 (100.0%)	56 (19.7%)	36 (100.0%)	32 (14.4%)
	**neg (%)**	19 (47.5%)	209 (79.2%)	0 (0.0%)	228 (80.3%)	0 (0.0%)	190 (85.6%)

Abbreviation: *pos* positive, *neg* negative, *PCR* Polymerase chain reaction.

There were 17 patients that were positive both for routine and research blood slides, while three of the positive research slides were negative on routine microscopy. The average parasitaemia among all positive research slides was 3.03% infected red blood cells (RBCs) or 151,500 parasites/μl with all having > 0.1% infected RBCs or > 5,000 parasites/μl. For the routine slides the average parasitaemia was 355 parasites per 200 white blood cells or 19,044 parasites/μl. There was statistically significant association between high parasitaemia (>10,000 parasites/μl) on routine slides and both positivity on research slide and positivity on PCR (p < 0.001). Only one routine slide with high parasitaemia was negative by all other diagnostics methods while remaining false positive routine slides were reported as having low parasitaemia.

Of the 258 cases where RDTs were performed, 36 were positive. Both HRP2 (*P. falciparum*) and pLDH (PAN) was positive for 25 of these, ten were positive only for HRP2 and one was only pLDH-positive. Two tests were invalid (no control band), but were valid when repeated. Among those positive by routine microscopy, only half of them were also positive by RDT and most of these (17/18) were positive by both pLDH and HRP2. All those positive on research blood slides were positive for both pLDH and HRP2. Of those positive by RDT, all were also positive on species-specific PCR for *P. falciparum.* There was a significant association between signs of severe malaria (altered consciousness, severe anaemia, jaundice or respiratory distress) and positive RDT (p < 0.001). No significant association was found between positive PCR or positive research blood smear and signs of severe malaria. There was no significant association between high parasitaemia on routine or research blood smear and signs of severe malaria.

The sensitivities, specificities, number of false positives and false negatives are given in Additional file 2. These values were calculated using the PCR result, research thin blood smear and RDT results separately as gold standards.

Comparisons with results of the mitochondrial PCR, which has a very high sensitivity [22], research microscopy and RDT results indicate that routine microscopy results in high numbers of false positives. Thus, the routine microscopy results were not included in further analysis of attributable morbidity and mortality.

### Univariate analysis

Results of analysis comparing cases with positive research blood slide with cases with negative research blood slide, PCR-positive cases with PCR-negative cases, and cases with positive RDT with cases with negative RDT are shown in Additional file 1. Levels of leucocytes, neutrophils, monocytes and lymphocytes were not significantly associated with positive PCR or research blood slide.

The cases only positive by PCR but with negative research blood slide examination are compared to cases positive by both PCR and research blood slide in Table 2.

**Table 2:** Univariate analysis of predictors of negative research blood slide among PCR positive cases

**Characteristic**	**PCR pos only (%)**	**OR (95% CI)**	**p-value**
***Demographics;***			
*•Age > 12 months*	34/52 (65.4)	0.18 (0.04 to 0.86)	0.019*
*•Male*	29/41 (70.7)	0.72 (0.25 to 2.02)	0.527
*•Very low weight for age*	7/11 (63.6)	0.60 (0.15 to 2.33)	0.474
*•Mothers education less than secondary school*	48/64 (75.0)	1.50 (0.34 to 6.70)	0.688
*•No mosquito net used*	4/5 (80.0)	1.58 (0.17 to 15.19)	1.000
*•Travel outside Dar last 4 weeks*	21/30 (70.0)	0.84 (0.28 to 2.50)	0.754
*•Home not in Dar*	5/8 (62.5)	0.43 (0.09 to 2.10)	0.365
*•Sickle cell disease*	12/18 (66.7)	0.64 (0.20 to 2.01)	0.542
*•Referral from other hospital*	14/19 (73.7)	0.98 (0.30 to 3.22)	1.000
***Pretreatment:***			
*•No antibiotics the last 4 weeks*	13/21 (61.9)	0.28 (0.09 to 0.94)	0.055
*•No antimalarials the last 4 weeks*	20/27 (74.1)	0.98 (0.33 to 2.95)	0.974
***Symptoms:***			
*•Current illness ≤5 days*	29/47 (61.7)	0.06 (0.01 to 0.50)	0.001*
*•Convulsions before admission*	13/17 (76.5)	1.21 (0.34 to 4.26)	1.000
***Clinical findings:***			
*•Reduced conciousness*	18/25 (72.0)	0.90 (0.31 to 2.65)	0.854
*•Tachycardia, for age*	22/27 (81.5)	2.12 (0.67 to 6.79)	0.199
*•Tachypnea, for age*	31/35 (88.6)	5.39 (1.59 to 18.28)	0.004*
*•Febrile (temp >37.5)*	40/56 (71.4)	0.71 (0.20 to 2.50)	0.764
*•Palmar pallor*	40/57 (70.2)	0.21 (0.026 to 1.79)	0.163
*•Jaundice*	13/15 (86.7)	2.72 (0.56 to 13.30)	0.328
*•Splenomegaly*	9/12 (75.0)	1.09 (0.27 to 4.49)	1.000
*•Hepatomegaly*	24/33 (72.7)	0.91 (0.33 to 2.56)	0.868
*•Abdominal distention*	12/16 (75.0)	1.09 (0.31 to 3.88)	1.000
***Laboratory findings:***			
*•Low Hb (<9.0 g/dl)*	43/61 (70.5)	0.37 (0.08 to 1.80)	0.328
*•Platelets <100 x10*^*3*^*per mm*^*3*^	11/18 (61.1)	0.49 (0.16 to 1.51)	0.233
*•Leucocytosis for age*	19/21 (90.5)	4.62 (0.97 to 22.04)	0.040*
***Treatment in hospital:***			
*•No antibiotic treatment in hospital*	3/6 (50.0)	0.32 (0.06 to 1.74)	0.183
*•Anti-malarial treatment in hospital*	46/66 (69.7)		0.055
*•Blood transfusion given*	18/29 (62.1)	0.39 (0.14 to 1.10)	0.071
***Results:***			
*•Blood culture negative*	11/15 (73.3)	0.79 (0.11 to 5.49)	1.000
*•routine malaria slide positive*	4/21 (19.0)	0.01 (<0.01 to 0.07)	<0.001*
*•high parasitaemia (>10,000 parasites/*μ*l), routine slide*	1/15 (6.7)	0.07 (0.01 to 0.95)	0.053
*•Received diagnosis malaria*	28/43 (65.1)	0.33 (0.11 to 1.04)	0.053
*•Length of admission ≤5 days*	33/44 (75.0)	1.17 (0.42 to 3.29)	0.760
*•Died in hospital*	12/15 (80.0)	1.55 (0.39 to 6.16)	0.746

Abbreviation: *OR* Odds ratio, *95% CI* 95% Confidence interval, *Dar* Dar es Salaam, *pos* positive, *PCR* Polymerase chain reaction, *Hb* Haemoglobin. * significant results (p-value < 0.05).

### Multivariate analysis

In the logistic regression model using PCR result as the outcome variable, the variables significantly associated with a positive result are shown in Additional file 3, as are the results of the corresponding models performed with research blood slide results as outcome variable and RDT result as outcome variable. In the logistic regression model, no factors were significantly associated with only being PCR positive (but research slide negative) compared to those also research slide positive.

**Figure 1:**
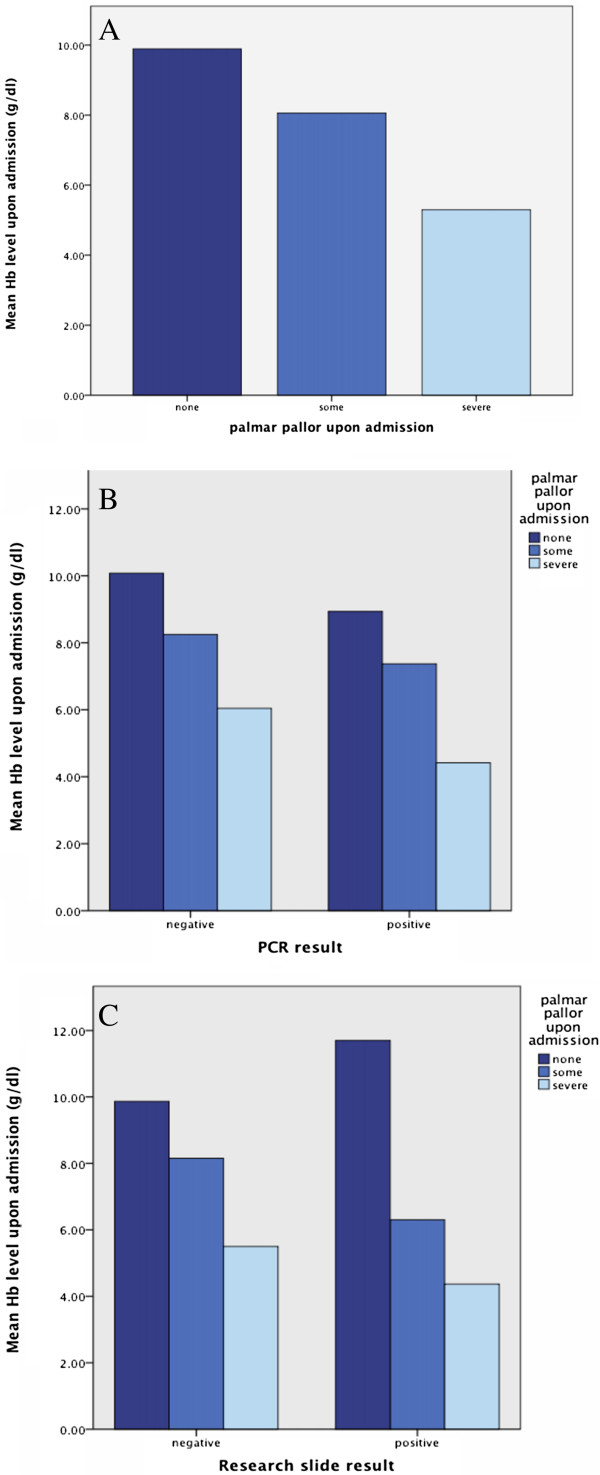
**Relation between palmar pallor and haemoglobin level. A**. For all cases. **B**. Based on PCR results. **C**. Based on research slide results.

Palmar pallor, as reported by the clinicians, corresponded well to low haemoglobin levels with the same trend applying when analysing the PCR positive and the study blood smear positive cases separately (Figure 1).

Death during hospital stay was significantly associated with reduced consciousness upon admission (p < 0.001), convulsions before admission (p = 0.027), lower age (p < 0.001), shorter hospital stay (p < 0.001), and hepatomegaly (p = 0.003). Sickle cell disease was significantly more frequent among those who survived compared to the children who died in hospital (p < 0.001).

## Discussion

The study assessed children admitted at MNH with febrile illness for malaria, and compared laboratory results to clinical and haematological features and clinical outcome.

Almost half of the cases identified by routine microscopy at the hospital were negative on research microscopy, RDT and PCR. The study thus confirms that routine malaria diagnosis leads to gross overdiagnosis of malaria, as was reported from other health facilities in Dar es Salaam in a previous study [25]. In both studies, false positive routine microscopy was associated with reported low parasitaemia. Conversely, the majority (85%, n = 17) of the samples positive on research microscopy were also positive on routine microscopy and these routine slides had a high average parasitaemia (>10,000 parasites/μl). This indicates that, despite the large problem with overdiagnosis, the routine thick-drop microscopy performs well in detecting malaria cases with high parasitaemia, which are those most likely to be severely ill and in need of urgent treatment. The fact that thick drop smears are more difficult to interpret because of lacking red cell morphology and many artefacts may partially explain the high rate of false positives [26].

The study confirmed that palmar pallor, low haemoglobin levels and low platelets are predictors for malaria [27,28]. Anaemia is often a sensitive, though non-specific indicator of malaria in febrile children in malaria-endemic areas [29,30], and the combination of anaemia and thrombocytopaenia has been shown to be fairly sensitive (80%) and specific (84%) in predicting malaria [29]. The study confirmed the usefulness of palmar pallor as a clinical sign of anaemia and severity of anaemia [31], and this clinical sign is particularly valuable in resource-constrained settings where laboratory testing for haemoglobin may not be available. Severe palmar pallor was particularly accurate in identifying severe anaemia with haemoglobin levels < 6.0 g/dl, which is associated with severe malaria [32].

PCR was positive in a fifth of patients with negative microscopy. The PCR method used in this study has nearly 100% sensitivity and specificity for the presence of malaria DNA [22] and all results were validated by species-specific PCR or DNA sequencing. PCR-positive patients may represent unidentified clinical malaria cases requiring anti-malarial treatment or low parasitaemia without clinical significance for example from partially or fully treated cases. The PCR targets the mitochondrial genome, which exists at a high copy number particularly in gametocytes due to an increased number of mitochondrial organelles in the parasite′s sexual stage [33], and may therefore detect low levels of persisting asexual parasites or gametocytes. Gametocytes may survive anti-malarial treatment with ACT and continue to circulate more than 28 days after completed treatment, without any clinical symptoms [34,35]. Though reported pre-treatment with anti-malarials was not significantly associated with positive PCR for malaria, self-treatment with anti-malarials before seeking help from health services is so common practice that it may have influenced the results [36,37]. Asymptomatic malaria, though less common in children than in adults, may cause positive PCR results in febrile children with other causes of fever than malaria, while microscopy might not detect this low level parasitemia [38-40]. Younger children, in the current study, had an increased risk of discordant results with positive PCR and negative research blood slide, which can be explained by an observed higher rate of prior anti-malarial treatment. The clinical relevance of the positive PCR in microscopy-negative patients remains unclear. Thus, as it is not possible to identify a true gold standard for malaria in the current study, the true incidence of malaria in the population remains uncertain. The new recommendations from WHO in 2010 to only treat laboratory-confirmed malaria cases if laboratory diagnostics are available, rather than to treat all suspected paediatric cases with anti-malarials [15], has reemphasized the importance of precise diagnostics.

RDTs have shown promising results as an alternative to microscopy for malaria, though they have certain limitations especially due to the fact that RDTs detecting HRP2 can remain positive for up to 56 days after completed treatment [41]. Re-infections are difficult to identify, resulting in a low specificity of HRP2-detecting RDTs for true clinical infection. This challenge is highly relevant in the study area, which is located in a malaria endemic region. The other test antigen, pLDH, is cleared more quickly from the bloodstream at about the same time as the parasites are cleared. While rapid tests detecting pLDH are less sensitive than HRP2 tests [42], they have the advantage of also detecting other species of *Plasmodium* than *P. falciparum*. With implementation of a HRP2/pLDH RDT in the study area, some cases of non-falciparum malaria might go undetected. However, as *P. falciparum* dominates in the area and is the most dangerous of the *Plasmodium* species, RDTs are likely to be sensitive for the clinically most significant malaria cases. As most of the rapid tests were positive for both HRP2 and pLDH it can be assumed they were current *P. falciparum* cases. The one test that was only pLDH-positive was positive for *P. falciparum* on species-specific PCR and negative on the other species-specific PCRs and it can therefore be assumed to be a *P. falciparum* case despite the RDT result. The cases only positive for HRP2 may be cases with persisting HRP2 post-treatment or may be current infections as detection of pLDH is less sensitive than HRP2. Discordant, pLDH-negative / HRP2-positive, samples corresponded well to PCR-positive / microscopy-negative samples. This implies that malaria detected only on PCR may represent partially or fully treated cases of malaria of less clinical significance than samples detected by microscopy.

Positive RDT results, all confirmed by PCR, were associated with signs of severe malaria, while malaria cases diagnosed primarily by PCR or research blood slide microscopy were not. This may support the notion that the RDTs identify more relevant cases of malaria, confirming their usefulness in this clinical setting.

Studies have shown that with reduced use of anti-malarials due to better precision of malaria diagnostics, antibiotic use increases [43], and this may, in turn contribute to further emergence of antibiotic resistance [44]. In order to reduce overuse of drugs, improved diagnostics for malaria must been accompanied by better diagnostics for other infections.

The high case fatality rate of 22,7% among febrile children is striking and is likely due to transfer of many severely ill patients from district hospitals, and the fact that less severe cases were treated as outpatients and not included in the study. The similar case fatality rates between malaria positive and negative cases imply that there are other major causes of life-threatening febrile illness, such as bacteraemia, pneumonia, and meningitis in the study population. A previous study of febrile children at the same hospital observed a similarly high case fatality rate for malaria (20.2%), but still found that bacterial and fungal bloodstream infections caused more deaths than malaria [45]. The current study found a much lower case fatality rate among patients with positive research microscopy (4.3%) than those with positive PCR (20.7%). This might be explained by increased number of malaria deaths among untreated patients with false negative malaria microscopy. However, considering that a positive malaria PCR was not associated with clinical signs of severe malaria, the appropriate interpretation may be that a positive malaria-PCR in absence of other positive malaria tests indicates clinically non-significant malaria parasitaemia in a patient with another, potentially serious, infection.

Tachypnea, a feature of the systemic inflammatory response syndrome [46], was negatively associated with research microscopy-positive cases, but positively associated with discordant, PCR positive / microscopy negative, cases further suggesting that the real cause of the febrile illness in patients with discordant results is bacterial sepsis or other non-malarial infection.

The study area/population being an urban population with lower malaria transmission than the surrounding rural areas [9], may limit the applicability of the results to the general Tanzanian population. Both clinical and laboratory findings may be biased by the fact that many of the children had received prior treatment with antibiotics and anti-malarials and that two-thirds were referred from other hospitals or clinics. Since the study did not investigate systematically other causes of febrile illness, it is uncertain how many of the identified malaria cases had concomitant serious infections caused by bacteria or other agents.

## Conclusions

The study indicates that the current routine diagnostic method, while efficient at detecting high malaria parasitaemia, leads to gross overdiagnosis of malaria and, consequently, overuse of anti-malarials. Overdiagnosis of malaria may hamper the identification and treatment of the real cause of the febrile illness and lead to death from bacterial sepsis and other infections. The diagnostic challenges understandably make clinicians opt to safeguard using both anti-malarials and antibacterial drugs, however, this has potential harmful consequences in terms of increasing emerging antibiotic and anti-malarial drug resistance. The current study supports the notion that diagnostic algorithms employing RDTs may contribute to improving malaria diagnosis and management.

## Competing interests

The authors declare that they have no competing interests.

## Authors’ contributions

GEAS and BB were involved in all stages of this study. MRF participated in the coordination of the fieldwork. NL was involved in the design of the study. CH was involved in the laboratory work and development and optimization of the PCR-method. All authors contributed to the data interpretation and writing of the manuscript. All authors have read and approved the final manuscript.

## Supplementary Material

Additional file 1 Univariate analysis of predictors of positive malaria results by PCR, blood smear and RDT. Click here for file

Additional file 2 Sensitivity, specificity, false positive rate and false negative rate for the various diagnostic methods. Click here for file

Additional file 3 Logistic regression of predictors of positive PCR or positive blood slide or positive. Click here for file

## References

[B1] Liu L Johnson HL Cousens S Perin J Scott S Lawn JE Rudan I Campbell H Cibulskis R Li M Mathers C Black RE Child Health Epidemiology Reference Group of WHO and UNICEF Global, regional, and national causes of child mortality: an updated systematic analysis for 2010 with time trends since 2000 Lancet 2012 379 2151 2161 10.1016/S0140-6736(12)60560-1 22579125

[B2] WHO World Malaria Report 2012 2012 Geneva: World Health Organization

[B3] Reyburn H Mbatia R Drakeley C Carneiro I Mwakasungula E Mwerinde O Saganda K Shao J Kitua A Olomi R Greenwood BM Whitty CJ Overdiagnosis of malaria in patients with severe febrile illness in Tanzania: a prospective study BMJ 2004 329 1212 10.1136/bmj.38251.658229.55 15542534 PMC529364

[B4] Mwangi TW Mohammed M Dayo H Snow RW Marsh K Clinical algorithms for malaria diagnosis lack utility among people of different age groups Trop Med Int Health 2005 10 530 536 10.1111/j.1365-3156.2005.01439.x 15941415 PMC3521057

[B5] Scott JA Berkley JA Mwangi I Ochola L Uyoga S Macharia A Ndila C Lowe BS Mwarumba S Bauni E Marsh K Williams TN Relation between falciparum malaria and bacteraemia in Kenyan children: a population-based, case–control study and a longitudinal study Lancet 2011 378 1316 1323 10.1016/S0140-6736(11)60888-X 21903251 PMC3192903

[B6] Derua YA Ishengoma DR Rwegoshora RT Tenu F Massaga JJ Mboera LE Magesa SM Users’ and health service providers’ perception on quality of laboratory malaria diagnosis in Tanzania Malar J 2011 10 78 10.1186/1475-2875-10-78 21470427 PMC3084175

[B7] Rakotonirina H Barnadas C Raherijafy R Andrianantenaina H Ratsimbasoa A Randrianasolo L Jahevitra M Andriantsoanirina V Menard D Accuracy and reliability of malaria diagnostic techniques for guiding febrile outpatient treatment in malaria-endemic countries Am J Trop Med Hyg 2008 78 217 221 18256418

[B8] Mmbando BP Vestergaard LS Kitua AY Lemnge MM Theander TG Lusingu JP A progressive declining in the burden of malaria in north-eastern Tanzania Malar J 2010 9 216 10.1186/1475-2875-9-216 20650014 PMC2920289

[B9] Murray CJ Rosenfeld LC Lim SS Andrews KG Foreman KJ Haring D Fullman N Naghavi M Lozano R Lopez AD Global malaria mortality between 1980 and 2010: a systematic analysis Lancet 2012 379 413 431 10.1016/S0140-6736(12)60034-8 22305225

[B10] Ochola LB Vounatsou P Smith T Mabaso ML Newton CR The reliability of diagnostic techniques in the diagnosis and management of malaria in the absence of a gold standard Lancet Infect Dis 2006 6 582 588 10.1016/S1473-3099(06)70579-5 16931409

[B11] d’Acremont V Malila A Swai N Tillya R Kahama-Maro J Lengeler C Genton B Withholding antimalarials in febrile children who have a negative result for a rapid diagnostic test Clin Infect Dis 2010 51 506 511 10.1086/655688 20642354

[B12] O’Brien C Henrich PP Passi N Fidock DA Recent clinical and molecular insights into emerging artemisinin resistance in *Plasmodium falciparum* Curr Opin Infect Dis 2011 24 570 577 10.1097/QCO.0b013e32834cd3ed 22001944 PMC3268008

[B13] Singh B Bobogare A Cox-Singh J Snounou G Abdullah MS Rahman HA A genus- and species-specific nested polymerase chain reaction malaria detection assay for epidemiologic studies Am J Trop Med Hyg 1999 60 687 692 10348249 10.4269/ajtmh.1999.60.687

[B14] Morassin B Fabre R Berry A Magnaval JF One year’s experience with the polymerase chain reaction as a routine method for the diagnosis of imported malaria Am J Trop Med Hyg 2002 66 503 508 12201583 10.4269/ajtmh.2002.66.503

[B15] WHO Guidelines for the treatment of malaria 2010 2 Geneva: World Health Organization 25473692

[B16] Wang SJ Lengeler C Mtasiwa D Mshana T Manane L Maro G Tanner M Rapid Urban Malaria Appraisal (RUMA) II: epidemiology of urban malaria in Dar es Salaam (Tanzania) Malar J 2006 5 28 10.1186/1475-2875-5-28 16584575 PMC1489940

[B17] WHO World Malaria Report 2011 2011 Geneva: World Health Organization

[B18] Tanzania Commission for AIDS (TACAIDS) ZACZ, National Bureau of Statistics (NBS), Office of the Chief Government Statistician (OCGS), and ICF International Tanzania HIV/AIDS and Malaria Indicator Survey 2011–2012 2013 Dar es Salaam, Tanzania: TACAIDS, ZAC, NBS, OCGS, and ICF International

[B19] Unicef/WHO Handbook: IMCI integrated management of childhood illness 2006 Geneva: World Health Organization

[B20] WHO Pocket book of Hospital care for children 2005 Geneva: World Health Organization Press 24006557

[B21] Hanscheid T Diagnosis of malaria: a review of alternatives to conventional microscopy Clin Lab Haematol 1999 21 235 245 10.1046/j.1365-2257.1999.00220.x 10583325

[B22] Haanshuus C Mohn S Morch K Langeland N Blomberg B Hanevik K A novel, single-amplification PCR targeting mitochondrial genome highly sensitive and specific in diagnosing malaria among returned travellers in Bergen, Norway Malar J 2013 12 26 10.1186/1475-2875-12-26 23336125 PMC3556099

[B23] Padley D Moody AH Chiodini PL Saldanha J Use of a rapid, single-round, multiplex PCR to detect malarial parasites and identify the species present Ann Trop Med Parasitol 2003 97 131 137 10.1179/000349803125002977 12803868

[B24] WHO Malaria Rapid Diagnostic Test Performances: results of WHO product testing of malaria RDTs: Round 4 (2012) 2012 Geneva: World Health Organization

[B25] Kahama-Maro J D’Acremont V Mtasiwa D Genton B Lengeler C Low quality of routine microscopy for malaria at different levels of the health system in Dar es Salaam Malar J 2011 10 332 10.1186/1475-2875-10-332 22047131 PMC3217957

[B26] Chiodini PL Moody AH Techniques for the detection of malaria parasites J R Soc Med 1989 82 Suppl 17 41 43 2693725 PMC1291939

[B27] Maina RN Walsh D Gaddy C Hongo G Waitumbi J Otieno L Jones D Ogutu BR Impact of *Plasmodium falciparum* infection on haematological parameters in children living in Western Kenya Malar J 2010 9 Suppl 3 S4 10.1186/1475-2875-9-S3-S4 21144084 PMC3002140

[B28] Lacerda MV Mourao MP Coelho HC Santos JB Thrombocytopenia in malaria: who cares? Mem Inst Oswaldo Cruz 2011 106 Suppl 1 52 63 21881757 10.1590/s0074-02762011000900007

[B29] Menendez C Fleming AF Alonso PL Malaria-related anaemia Parasitol Today 2000 16 469 476 10.1016/S0169-4758(00)01774-9 11063857

[B30] Premji Z Hamisi Y Shiff C Minjas J Lubega P Makwaya C Anaemia and *Plasmodium falciparum* infections among young children in an holoendemic area, Bagamoyo, Tanzania Acta Trop 1995 59 55 64 10.1016/0001-706X(94)00079-G 7785526

[B31] Zucker JR Perkins BA Jafari H Otieno J Obonyo C Campbell CC Clinical signs for the recognition of children with moderate or severe anaemia in western Kenya Bull World Health Organ 1997 75 Suppl 1 97 102 9529722 PMC2486997

[B32] WHO Management of the child with a serious infection or severe malnutrition: guidelines for care at the first-referral level in developing countries 2000 Geneva: World Health Organization

[B33] Krungkrai J The multiple roles of the mitochondrion of the malarial parasite Parasitology 2004 129 Pt 5 511 524 15552397 10.1017/s0031182004005888

[B34] Tjitra E Suprianto S McBroom J Currie BJ Anstey NM Persistent ICT malaria P.f/P.v panmalarial and HRP2 antigen reactivity after treatment of *Plasmodium falciparum* malaria is associated with gametocytemia and results in false-positive diagnoses of *Plasmodium vivax* in convalescence J Clin Microbiol 2001 39 1025 1031 10.1128/JCM.39.3.1025-1031.2001 11230422 PMC87868

[B35] Schneider P Bousema T Omar S Gouagna L Sawa P Schallig H Sauerwein R (Sub)microscopic *Plasmodium falciparum* gametocytaemia in Kenyan children after treatment with sulphadoxine-pyrimethamine monotherapy or in combination with artesunate Int J Parasitol 2006 36 403 408 10.1016/j.ijpara.2006.01.002 16500657

[B36] McCombie SC Treatment seeking for malaria: a review of recent research Soc Sci Med 1996 43 933 945 10.1016/0277-9536(95)00446-7 8888463

[B37] Hetzel M Dillip A Lengeler C Obrist B Msechu J Makemba A Mshana C Schulze A Mshinda H Malaria treatment in the retail sector: Knowledge and practices of drug sellers in rural Tanzania BMC Publ Health 2008 8 157 10.1186/1471-2458-8-157 PMC2405791 18471299

[B38] Nkoghe D Akue JP Gonzalez JP Leroy EM Prevalence of *Plasmodium falciparum* infection in asymptomatic rural Gabonese populations Malar J 2011 10 33 10.1186/1475-2875-10-33 21306636 PMC3041723

[B39] Bousema J Gouagna L Drakeley C Meutstege A Okech B Akim I Beier J Githure J Sauerwein R *Plasmodium falciparum* gametocyte carriage in asymptomatic children in western Kenya Malar J 2004 3 18 10.1186/1475-2875-3-18 15202944 PMC441400

[B40] Wongsrichanalai C Barcus MJ Muth S Sutamihardja A Wernsdorfer WH A review of malaria diagnostic tools: microscopy and rapid diagnostic test (RDT) Am J Trop Med Hyg 2007 77 6 Suppl 119 127 18165483

[B41] Kyabayinze DJ Tibenderana JK Odong GW Rwakimari JB Counihan H Operational accuracy and comparative persistent antigenicity of HRP2 rapid diagnostic tests for *Plasmodium falciparum* malaria in a hyperendemic region of Uganda Malar J 2008 7 221 10.1186/1475-2875-7-221 18959777 PMC2584069

[B42] Hopkins H Kambale W Kamya MR Staedke SG Dorsey G Rosenthal PJ Comparison of HRP2- and pLDH-based rapid diagnostic tests for malaria with longitudinal follow-up in Kampala, Uganda Am J Trop Med Hyg 2007 76 1092 1097 17556616

[B43] D’Acremont V Kahama-Maro J Swai N Mtasiwa D Genton B Lengeler C Reduction of anti-malarial consumption after rapid diagnostic tests implementation in Dar es Salaam: a before-after and cluster randomized controlled study Malar J 2011 10 107 10.1186/1475-2875-10-107 21529365 PMC3108934

[B44] Barie PS Multidrug-resistant organisms and antibiotic management Surg Clin North Am 2012 92 345 391 ix-x 10.1016/j.suc.2012.01.015 22414417

[B45] Blomberg B Manji KP Urassa WK Tamim BS Mwakagile DS Jureen R Msangi V Tellevik MG Holberg-Petersen M Harthug S Maselle SY Langeland N Antimicrobial resistance predicts death in Tanzanian children with bloodstream infections: a prospective cohort study BMC Infect Dis 2007 7 43 10.1186/1471-2334-7-43 17519011 PMC1891109

[B46] Bone RC Sepsis, sepsis syndrome, and the systemic inflammatory response syndrome (SIRS). Gulliver in Laputa JAMA 1995 273 155 156 10.1001/jama.1995.03520260077036 7799497

