# Supplementary material for: Challenges in diagnosing paediatric malaria in Dar es Salaam, Tanzania
Source: Malar J. 2013 Jul 3;12:228. doi: 10.1186/1475-2875-12-228 (PMC3703277; doi:10.1186/1475-2875-12-228)
Supplement: Additional file 1 — Univariate analysis of predictors of positive malaria results by PCR, blood smear and RDT. [file 1475-2875-12-228-S1.docx]

Additional file 1: Univariate analysis of predictors of positive malaria results by PCR, blood smear.and RDT

| **Characteristic** | **PCR pos (%)** | **OR (95% CI)** | **P-value** | **Research slide pos (%)** | **OR (95% CI)** | **P-value** | **RDT pos (%)** | **OR (95% CI)** | **P-value** |
| --- | --- | --- | --- | --- | --- | --- | --- | --- | --- |
| ***Demographics*** |  |  |  |  |  |  |  |  |  |
| - *Age > 12 months* | 52/155 (33.5) | 2.68 (1.54 to 4.67) | <0.001* | 18/155 (11.6) | 9.39 (2.14 to 41.25) | <0.001* | 30/131 (22.9) | 5.79 (2.32 to 14.48) | <0.001* |
| - *Male* | 41/175 (23.4) | 0.82 (0.49 to 1.39) | 0.461 | 12/175 (6.9) | 1.11 (0.44 to 2.81) | 0.820 | 18/145 (12.4) | 0.75 (0.37 to 1.51) | 0.419 |
| - *Very low weight for age* | 11/69 (15.9) | 0.44 (0.22 to 0.90) | 0.022* | 4/69 (5.8) | 0.74 (0.24 to 2.32) | 0.788 | 6/54 (11.1) | 0.62 (0.24 to 1.58) | 0.308 |
| - *Mother education lower than secondary school* | 64/217 (29.5) | 2.37 (1.10 to 5.10) | 0.024* | 16/217 (7.4) | 1.51 (0.43 to 5.37) | 0.773 | 30/185 (16.2) | 2.23 (0.75 to 6.65) | 0.143 |
| - *No mosquito net used* | 5/9 (55.6) | 4.00 (1.04 to 15.37) | 0.045* | 1/9 (11.1) | 1.73 (0.20 to 14.63) | 0.480 | 3/7 (42.9) | 5.06 (1.08 to 23.82) | 0.057 |
| - *Travel outside Dar last 4 weeks* | 30/66 (45.5) | 3.87 (2.10 to 7.13) | <0.001* | 9/66 (13.6) | 3.21 (1.22 to 8.47) | 0.022* | 19/55 (34.5) | 6.77 (3.02 to 15.21) | <0.001* |
| - *Home not in Dar* | 8/32 (25.0) | 1.08 (0.46 to 2.56) | 0.858 | 3/32 (9.4) | 2.05 (0.53 to 7.88) | 0.390 | 5/20 (20.0) | 1.76 (0.60 to 5.17) | 0.298 |
| - *Sickle cell disease* | 18/46 (39.1) | 2.21 (1.14 to 4.27) | 0.017* | 6/46 (13.0) | 2.60 (0.95 to 7.17) | 0.097 | 9/43 (20.9) | 1.83 (0.79 to 4.23) | 0.152 |
| - *Referral from other hospital* | 19/93 (20.4) | 0.62 (0.34 to 1.12) | 0.112 | 5/93 (5.4) | 0.69 (0.24 to 1.98) | 0.488 | 6/80 (7.5) | 0.35 (0.14 to 0.87) | 0.020* |
| ***Pretreatment:*** |  |  |  |  |  |  |  |  |  |
| - *No antibiotics the last 4 weeks* | 21/70 (30.0) | 1.43 (0.78 to 2.63) | 0.245 | 8/70 (11.4) | 3.63 (1.27 to 10.42) | 0.027* | 14/61 (23.0) | 2.92 (1.33 to 6.43) | 0.006* |
| - *No antimalarials the last 4 weeks* | 27/101 (26.7) | 1.07 (0.61 to 1.87) | 0.815 | 7/101 (6.9) | 1.07 (0.40 to 2.85) | 0.893 | 8/87 (9.2) | 0.47 (0.20 to 1.10) | 0.075 |
| ***Symptoms:*** |  |  |  |  |  |  |  |  |  |
| - *Current illness* ≤*5 days* | 47/175 (26.9) | 1.21 (0.70 to 2.09) | 0.492 | 18/175 (10.3) | 13.18 (1.74 to 100.2) | 0.001* | 26/149 (17.4) | 2.38 (1.03 to 5.50) | 0.038* |
| - *Convulsions before admission* | 17/68 (25.0) | 0.99 (0.53 to 1.84) | 0.971 | 4/68 (5.9) | 0.85 (0.28 to 2.64) | 1.000 | 9/55 (16.4) | 1.26 (0.56 to 2.87) | 0.580 |
| ***Clinical findings:*** |  |  |  |  |  |  |  |  |  |
| - *Reduced conciousness* | 25/75 (33.3) | 1.74 (0.98 to 3.09) | 0.057 | 7/75 (9.3) | 1.67 (0.64 to 4.36) | 0.290 | 13/60 (21.7) | 2.04 (0.96 to 4.34) | 0.059 |
| - *Tachycardia, for age* | 27/81 (33.3) | 1.74 (0.98 to 3.09) | 0.056 | 5/81 (6.2) | 0.84 (0.29 to 2.42) | 0.748 | 12/63 (19.0) | 1.59 (0.74 to 3.45) | 0.234 |
| - *Tachypnea, for age* | 35/150 (23.3) | 0.80 (0.47 to 1.36) | 0.417 | 4/150 (2.7) | 0.22 (0.07 to 0.66) | 0.004* | 16/128 (12.5) | 0.75 (0.37 to 1.54) | 0.435 |
| - *Febrile (temp* >*37.5)* | 56/242 (23.1) | 0.69 (0.37 to 1.29) | 0.239 | 16/242 (6.6) | 0.97 (0.31 to 3.03) | 1.000 | 24/203 (11.8) | 0.45 (0.21 to 0.97) | 0.038* |
| - *Palmar pallor* | 57/205 (27.8) | 2.05 (1.03 to 4.09) | 0.038* | 17/205 (8.3) | 6.78 (0.89 to 51.87) | 0.050 | 32/173 (18.5) | 7.38 (1.72 to 31.71) | 0.002* |
| - *Jaundice* | 15/36 (41.7) | 2.42 (1.18 to 4.99) | 0.014* | 2/36 (5.6) | 0.82 (0.18 to 3.68) | 1.000 | 7/32 (21.9) | 1.90 (0.76 to 4.79) | 0.176 |
| - *Splenomegaly* | 12/31 (38.7) | 1.89 (0.87 to 4.10) | 0.106 | 3/31 (9.7) | 1.45 (0.40 to 5.26) | 0.476 | 9/28 (32.1) | 3.56 (1.46 to 8.66) | 0.003* |
| - *Hepatomegaly* | 33/95 (34.7) | 1.90 (1.10 to 3.27) | 0.021* | 9/95 (9.5) | 1.67 (0.67 to 4.19) | 0.267 | 17/80 (21.2) | 2.26 (1.10 to 4.62) | 0.023* |
| - *Abdominal distention* | 16/43 (37.2) | 1.99 (1.00 to 3.93) | 0.046* | 4/43 (9.3) | 1.57 (0.50 to 4.94) | 0.502 | 6/34 (17.6) | 1.39 (0.53 to 3.63) | 0.594 |
| ***Laboratory findings:*** |  |  |  |  |  |  |  |  |  |
| - *Low Hb (<9.0 g/dl)* | 61/199 (30.7) | 2.59 (1.39 to 4.85) | 0.002* | 18/199 (9.0) | 5.02 (1.14 to 22.08) | 0.019* | 32/168 (19.0) | 4.94 (1.69 to 14.47) | 0.002* |
| - *Platelets <100 x103 per mm3* | 18/31 (58.1) | 5.38 (2.48 to 11.61) | <0.001* | 7/31 (22.6) | 5.72 (2.08 to 15.70) | 0.002* | 10/29 (34.5) | 4.19 (1.75 to 10.01) | 0.001* |
| - *Monocytes raised, for age* | 49/200 (24.5) | 0.89 (0.51 to 1.54) | 0.674 | 15/200 (7.5) | 1.56 (0.55 to 4.41) | 0.402 | 30/171 (17.5) | 2.77 (1.10 to 6.94) | 0.025* |
| ***Treatment in hospital:*** |  |  |  |  |  |  |  |  |  |
| - *No antibiotic treatment in hospital* | 6/12 (50.0) | 3.17 (0.99 to 10.15) | 0.080 | 3/12 (25.0) | 5.39 (1.34 to 21.77) | 0.037* | 2/10 (20.0) | 1.57 (0.32 to 7.73) | 0.635 |
| - *Antimalarial treatment in hospital* | 66/198 (33.3) | 4.80 (2.35 to 9.81) | <0.001* | 20/198 (10.1) | - | 0.001* | 34/162 (21.0) | 12.48 (2.93 to 53.26) | <0.001* |
| - *Blood transfusion given* | 29/60 (48.3) | 3.92 (2.16 to 7.13) | <0.001* | 11/60 (18.3) | 5.86 (2.31 to 14.90) | <0.001* | 22/54 (40.7) | 9.33 (4.33 to 20.10) | <0.001* |
| ***Results:*** |  |  |  |  |  |  |  |  |  |
| - *Routine malaria slide positive* | 21/40 (52.5) | 4.20 (2.11 to 8.36) | <0.001* | 17/40 (42.5) | 64.3 (17.54 to 235.8) | <0.001* | 18/36 (50.0) | 11.33 (5.03 to 25.52) | <0.001* |
| - *High parasitemia (>10 000 parasites/μl), routine slide* | 15/16 (93.8) | 45.00 (4.86 to 416.46) | <0.001* | 14/16 (87.5) | 49.0 (7.24 to 331.81) | <0.001* | 13/14 (92.9) | 44.20 (4.59 to 425.79) | <0.001* |
| - *Received diagnosis malaria* | 43/124 (34.7) | 2.33 (1.38 to 3.96) | 0.001* | 15/124 (12.1) | 4.76 (1.68 to 13.47) | 0.001* | 28/106 (26.4) | 6.37 (2.77 to 14.66) | <0.001* |
|  |  |  |  |  |  |  |  |  |  |
| - *Length of admission* ≤*5 days* | 44/153 (28.8) | 1.50 (0.89 to 2.54) | 0.128 | 11/153 (7.2) | 1.22 (0.49 to 3.04) | 0.666 | 21/132 (15.9) | 1.40 (0.69 to 2.86) | 0.353 |
| - *Died in hospital* | 15/69 (21.7) | 0.79 (0.42 to 1.51) | 0.477 | 3/69 (4.3) | 0.58 (0.17 to 2.05) | 0.582 | 6/58 (10.3) | 0.65 (0.26 to 1.66) | 0.368 |
| OR, odds ratio; 95% CI, 95% confidence interval; Dar, Dar es Salaam; pos, positive; PCR, polymerase chain reaction; Hb, hemoglobin; RDT, rapid diagnostic test for malaria. * significant results (p-value <0.05). | | | | | | | | | |
