# Supplementary material for: Challenges in diagnosing paediatric malaria in Dar es Salaam, Tanzania
Source: Malar J. 2013 Jul 3;12:228. doi: 10.1186/1475-2875-12-228 (PMC3703277; doi:10.1186/1475-2875-12-228)
Supplement: Additional file 2 — Sensitivity, specificity, false positive rate and false negative rate for the various diagnostic methods. [file 1475-2875-12-228-S2.docx]

Additional file 2: Sensitivity, specificity, false positive rate and false negative rate for the various diagnostic methods

|  | **PCR as gold standard** | | | | **Study microscopy as gold standard** | | | | **RDT as gold standard** | | | |
| --- | --- | --- | --- | --- | --- | --- | --- | --- | --- | --- | --- | --- |
|  | Sens | Spec | False pos | False neg | Sens | Spec | False pos | False neg | Sens | Spec | False pos | False neg |
| **Routine micro** | 27.6% | 91.7% | 8.3% | 72.4% | 85.0% | 91.9% | 8.1% | 15.0% | 50.0% | 91.9% | 8.1% | 50.0% |
| **Study micro** | 26.3% | 100.0% | 0% | 73.7% | - | - | - | - | 100.0% | 91.7% | 8.3% | 0.0% |
| **PCR** | - | - | - | - | 100.0% | 80.3% | 19.7% | 0% | 100.0% | 85.6% | 14.4% | 0.0% |
| **RDT** | 52.9% | 100.0% | 0% | 47.1% | 100.0% | 86.5% | 7.8% | 0% | - | - | - | - |
| PCR, polymerase chain reaction; RDT, rapid diagnostic test for malaria; micro, microscopy; Sens, sensitivity; Spec, specificity; pos, positive; neg, negative. | | | | | | | | | | | | |
