# Supplementary material for: Challenges in diagnosing paediatric malaria in Dar es Salaam, Tanzania
Source: Malar J. 2013 Jul 3;12:228. doi: 10.1186/1475-2875-12-228 (PMC3703277; doi:10.1186/1475-2875-12-228)
Supplement: Additional file 3 — Logistic regression of predictors of positive PCR or positive blood slide or positive. [file 1475-2875-12-228-S3.docx]

Additional file 3: Logistic regression of predictors of positive PCR or positive blood slide or positive RDT

| **Characteristic** | **PCR pos (N=219)** | **Research slide pos (N=218)** | **RDT pos (N=190)** |
| --- | --- | --- | --- |
|  | OR (95% CI) | OR (95% CI) | OR (95% CI) |
| ***Demographics:*** |  |  |  |
| - *Age > 12 months* | 2.56 (1.22-5.37)† | 9.29 (1.72-50.18)† | 6.91 (1.47-26.11)† |
| - *Male* | * | * | * |
| - *Very low weight for age* | 0.43 (0.17-1.08) | * | - |
| - *Mothers education less than secondary school* | 4.33 (1.53-12.25)† | * | * |
| - *No mosquito net used* | 3.81 (0.72-20.07) | * | 10.83 (1.23-95.63)† |
| - *Travel outside Dar last 4 weeks* | 4.55 (2.09-9.91)‡ | 5.18 (1.45-18.58)† | 44.02 (8.79-220.52)‡ |
| - *Sickle cell disease* | * | * | 3.56 (0.62-20.34) |
| - *Referral from other hospital* | * | 0.47 (0.13-1.78) | - |
| ***Pretreatment:*** |  |  |  |
| - *No antibiotics the last 4 weeks* | * | * | - |
| - *No antimalarials the last 4 weeks* | - | - | 0.16 (0.03-0.93)† |
| ***Symptoms:*** |  |  |  |
| - *Current illness ≤5 days* | * | 16.72 (1.94-143.74)† | 9.63 (1.93-41.13)† |
| ***Clinical findings:*** |  |  |  |
| - *Reduced conciousness* | 2.31 (0.94-5.67) | * | 10.30 (2.05 -51.90)† |
| - *Tachycardia for age* | * | * | - |
| - *No tachypnea for age* | * | 0.23 (0.06-0.94)† | - |
| - *Afebrile (temp ≤37.5)* | * | * | * |
| - *Jaundice* | * | 0.32 (0.05-1.93) | 0.10 (0.01-0.79)† |
| - *Splenomegaly* | * | * | * |
| - *Hepatomegaly* | 1.87 (0.86-4.04) | * | * |
| - *Abdominal distention* | * | * | * |
| ***Laboratory findings:*** |  |  |  |
| - *Low Hb (<9.0 g/dl)* | * | * | 3.82 (0.74-19.79) |
| - *Platelets <100 x103 per mm3* | 5.34 (1.78-16.04)† | * | 7.38 (1.10.49.53)† |
|  |  |  |  |
| - *Length of admission ≤5 days* | * | 0.34 (0.10-1.23) | * |
| - *Alive upon discharge* | 0.28 (0.07-1.07) | * | 0.22 (0.03-1.58) |
| OR, odds ratio; 95%CI, 95% confidence interval; Dar, Dar es Salaam; PCR, polymerase chain reaction; Hb, hemoglobin; RDT, rapid diagnostic test for malaria; pos, positive. – variables not included in the analyses because of too many missing values. * Variables with *P* > 0.2 were removed from the models. Statistically significant risk factors are marked † *P* < 0.05 and ‡ P<0.001. | | | |
